# Supplementary material for: Identification of Various InDel-II Variants of the White Spot Syndrome Virus Isolated from Frozen Shrimp and Bivalves Obtained in the Korean Commercial Market
Source: Animals (Basel). 2023 Oct 27;13(21):3348. doi: 10.3390/ani13213348 (PMC10650675; doi:10.3390/ani13213348)
Supplement: Supplementary file 1 [file animals-13-03348-s001.zip › Figure S1.pptx]

## Slide 1
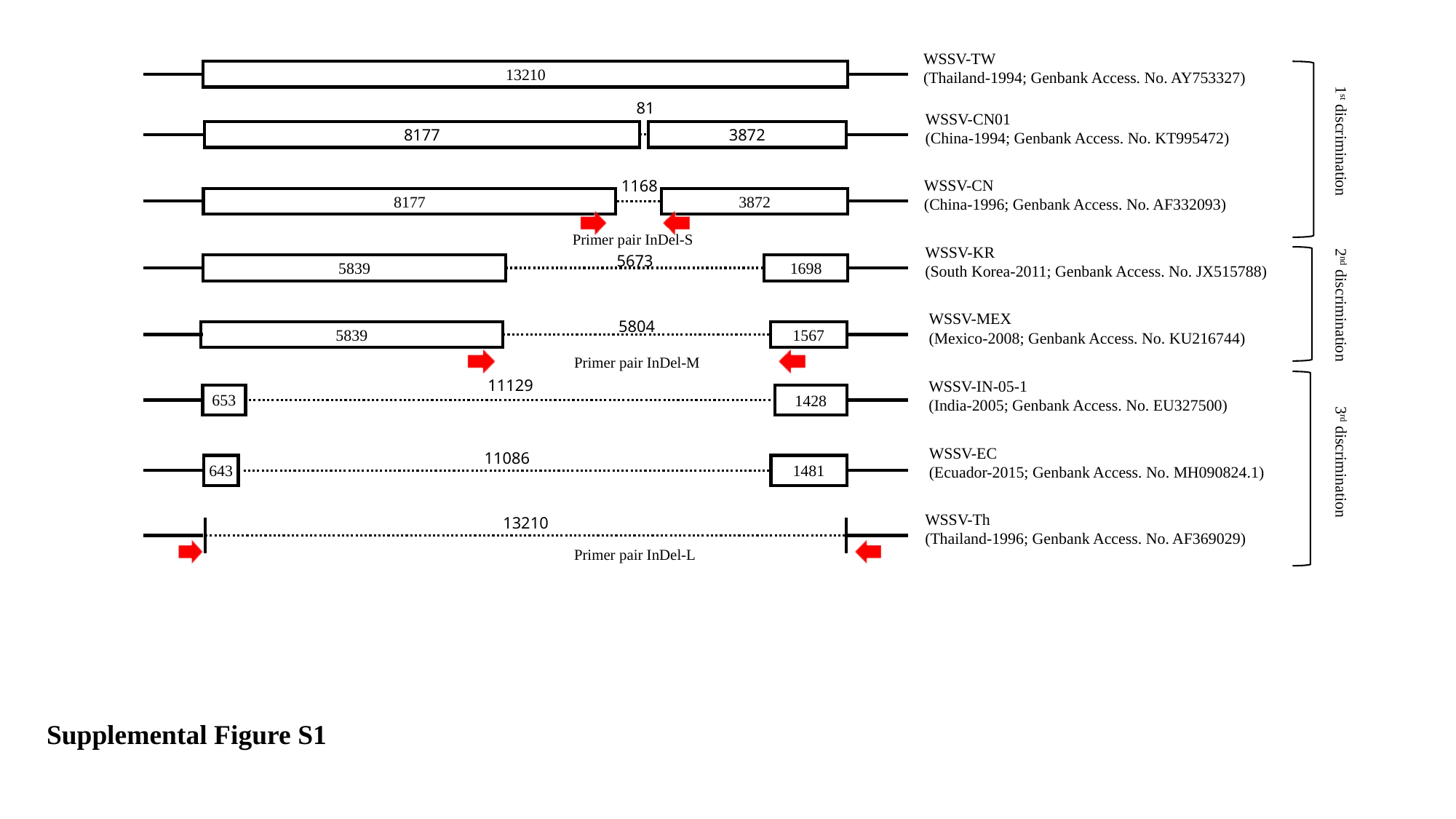

WSSV-TW
(Thailand-1994; Genbank Access. No. AY753327)
13210
81
WSSV-CN01
(China-1994; Genbank Access. No. KT995472)
8177
3872
1st discrimination
WSSV-CN
(China-1996; Genbank Access. No. AF332093)
1168
8177
3872
Primer pair InDel-S
WSSV-KR
(South Korea-2011; Genbank Access. No. JX515788)
5673
5839
1698
2nd discrimination
WSSV-MEX
(Mexico-2008; Genbank Access. No. KU216744)
5804
5839
1567
Primer pair InDel-M
11129
WSSV-IN-05-1
(India-2005; Genbank Access. No. EU327500)
653
1428
WSSV-EC
(Ecuador-2015; Genbank Access. No. MH090824.1)
11086
643
1481
3rd discrimination
WSSV-Th
(Thailand-1996; Genbank Access. No. AF369029)
13210
Primer pair InDel-L
Supplemental Figure S1
