# Supplementary material for: Identification of Various InDel-II Variants of the White Spot Syndrome Virus Isolated from Frozen Shrimp and Bivalves Obtained in the Korean Commercial Market
Source: Animals (Basel). 2023 Oct 27;13(21):3348. doi: 10.3390/ani13213348 (PMC10650675; doi:10.3390/ani13213348)
Supplement: Supplementary file 1 [file animals-13-03348-s001.zip › Table S1.pdf]

animals

MDPI

Supplemental Table S1. Summary of WSSV detection results in domestic and imported frozen shrimp and bivalves between 2011 and 2018

Sample Code[a]	Species	Country or region	Production time[b]	PCR[c]	qPCR[d]	InDel-II[e] (deletion length, bp)
**Domestic Shrimp**						
11-SA-3	*Litopenaeus vannamei*	Sinan	Mar.-2011	++	7.15×10^{6}	5,649
13-MP-7	*L. vannamei*	Mokpo	July-2013	++	9.92×10^{4}	5,649
13-GS-9	*L. vannamei*	Gosung	Sep.-2013	++	3.69×10^{5}	5,649
13-GH-6	*L. vannamei*	Goheung	Jun-2014	ND	NT	NT
14-SA-7	*L. vannamei*	Sinan	July-2014	+	3.77×10^{3}	11,070
14-TA-8	*L. vannamei*	Taean	Aug.-2014	ND	NT	NT
15-SC-6	*L. vannamei*	Seocheon	Jun-2015	ND	NT	NT
15-BR-7	*L. vannamei*	Boryeong	July-2015	++	3.28×10^{6}	11,070
15-YS-8	*L. vannamei*	Yeosu	Aug.-2015	+	7.91×10^{3}	13,046
15-MP-11	*L. vannamei*	Mokpo	Nov.-2015	ND	NT	NT
16-GC-8	*L. vannamei*	Gochang	Aug.-2016	+	2.24×10^{2}	5,649
16-GJ-7	*L. vannamei*	Geoje	July-2016	ND	NT	NT
16-SA-11	*L. vannamei*	Sinan	Nov.-2016	ND	NT	NT
17-BR-9	*L. vannamei*	Boryeong	Sep.-2017	+	4.14×10^{3}	11,070
17-TY-11	*L. vannamei*	Tongyeong	Nov.-2017	++	8.89×10^{6}	5,649
17-TA-11	*L. vannamei*	Taean	Nov.-2017	++	6.58×10^{6}	5,649
17-HS-12	*L. vannamei*	Hongseong	Dec.-2017	ND	NT	NT
18-BR-4	*L. vannamei*	Boryeong	Apr.-2018	ND	NT	NT
18-SA-10	*L. vannamei*	Sinan	Oct.-2018	++	7.26×10^{6}	777
**Imported shrimp**						
13-THA-1	*L. vannamei*	Thailand	Mar.-2013	++	1.46×10^{4}	ND
13-THA-2	*L. vannamei*	Thailand	Mar.-2013	ND	NT	NT
13-THA-3	*L. vannamei*	Thailand	Mar.-2013	+	3.24×10^{1}	ND
13-THA-4	*L. vannamei*	Thailand	May-2013	ND	NT	NT
13-THA-5	*L. vannamei*	Thailand	Jun-2013	ND	NT	NT
13-THA-6	*L. vannamei*	Thailand	Aug.-2013	ND	NT	NT
13-THA-7	*L. vannamei*	Thailand	Oct.-2013	ND	NT	NT
13-THA-8	*L. vannamei*	Thailand	Oct.-2013	ND	NT	NT
13-THA-9	*L. vannamei*	Thailand	Nov.-2013	+	ND	NT
14-THA-1	*L. vannamei*	Thailand	Apr.-2014	ND	NT	NT
14-THA-2	*L. vannamei*	Thailand	July-2014	ND	NT	NT
14-THA-3	*L. vannamei*	Thailand	July-2014	ND	NT	NT
15-THA-1	*L. vannamei*	Thailand	Apr.-2015	ND	NT	NT
15-THA-2	*L. vannamei*	Thailand	July-2015	+	1.07×10^{2}	13,210
15-THA-3	*L. vannamei*	Thailand	Aug.-2015	+	ND	NT
15-THA-4	*L. vannamei*	Thailand	Aug.-2015	+	1.39×10^{1}	ND
17-THA-1	*L. vannamei*	Thailand	Mar.-2017	+	ND	NT
17-THA-2	*L. vannamei*	Thailand	Sep.-2017	+	7.41×10^{1}	ND
18-THA-1	*L. vannamei*	Thailand	Jan.-2018	ND	NT	NT

13-MYS-1	*L. vannamei*	Malaysia	Feb.-2013	ND	NT	NT
13-MYS-2	*L. vannamei*	Malaysia	July-2013	+	ND	NT
13-MYS-3	*L. vannamei*	Malaysia	Aug.-2013	+	ND	NT
13-MYS-4	*L. vannamei*	Malaysia	Nov.-2013	ND	NT	NT
15-MYS-1	*L. vannamei*	Malaysia	July-2015	+	ND	NT
15-MYS-2	*L. vannamei*	Malaysia	July-2015	+	ND	NT
15-MYS-3	*L. vannamei*	Malaysia	Aug.-2015	+	8.15×10^{1}	ND
17-MYS-1	*L. vannamei*	Malaysia	Mar.-2017	+	3.03×10^{2}	ND
17-MYS-2	*L. vannamei*	Malaysia	Sep.-2017	+	9.72×10^{1}	ND
17-MYS-3	*L. vannamei*	Malaysia	Sep.-2017	ND	NT	NT
17-MYS-4	*L. vannamei*	Malaysia	Dec.-2017	ND	NT	NT
18-MYS-1	*L. vannamei*	Malaysia	Jan.-2018	ND	NT	NT
13-ECU-1	*L. vannamei*	Ecuador	July-2013	ND	NT	NT
13-ECU-2	*L. vannamei*	Ecuador	Sep.-2013	ND	NT	NT
14-ECU-1	*L. vannamei*	Ecuador	July-2014	+	ND	NT
15-ECU-1	*L. vannamei*	Ecuador	Mar.-2015	+	9.23×10^{1}	ND
15-ECU-2	*L. vannamei*	Ecuador	July-2015	+	ND	NT
15-ECU-3	*L. vannamei*	Ecuador	Aug.-2015	+	1.42×10^{2}	11,500
17-ECU-1	*L. vannamei*	Ecuador	Sep.-2017	ND	NT	NT
17-ECU-2	*L. vannamei*	Ecuador	Oct.-2017	+	7.72×10^{2}	11,086
17-ECU-3	*L. vannamei*	Ecuador	Dec.-2017	+	ND	NT
17-ECU-4	*L. vannamei*	Ecuador	Dec.-2017	+	ND	NT
18-ECU-1	*L. vannamei*	Ecuador	Jan.-2018	ND	NT	NT
18-ECU-2	*L. vannamei*	Ecuador	Apr.-2018	ND	NT	NT
13-IDN-1	*L. vannamei*	Indonesia	Feb.-2013	ND	NT	NT
13-IDN-2	*L. vannamei*	Indonesia	Jun-2013	ND	NT	NT
13-IDN-3	*L. vannamei*	Indonesia	July-2013	+	ND	NT
13-IDN-4	*L. vannamei*	Indonesia	Sep.-2013	+	ND	NT
13-IDN-5	*L. vannamei*	Indonesia	Nov.-2013	ND	NT	NT
14-IDN-1	*L. vannamei*	Indonesia	Apr.-2014	ND	NT	NT
14-IDN-2	*L. vannamei*	Indonesia	July-2014	+	NT	NT
15-IDN-1	*L. vannamei*	Indonesia	Apr.-2015	ND	NT	NT
15-IDN-2	*L. vannamei*	Indonesia	Jun-2015	+	1.84×10^{1}	ND
15-IDN-3	*L. vannamei*	Indonesia	Dec.-2015	ND	NT	NT
15-VNM-1	*L. vannamei*	Vietnam	Mar.-2015	ND	NT	NT
15-VNM-2	*L. vannamei*	Vietnam	July-2015	+	5.65×10^{1}	ND
17-VNM-1	*L. vannamei*	Vietnam	Mar.-2017	+	7.47×10^{2}	10,778
17-VNM-2	*L. vannamei*	Vietnam	Sep.-2017	ND	NT	NT
17-VNM-3	*L. vannamei*	Vietnam	Nov-2017	+	7.82×10^{1}	ND
18-VNM-1	*L. vannamei*	Vietnam	Apr-2018	+	ND	NT
13-ARG-1	*L. vannamei*	Argentina	Jun-2013	ND	NT	NT
14-ARG-1	*L. vannamei*	Argentina	July-2014	ND	NT	NT
15-ARG-1	*L. vannamei*	Argentina	July-2015	ND	NT	NT
17-ARG-1	*L. vannamei*	Argentina	Dec.-2017	ND	NT	NT
15-SAU-1	*L. vannamei*	Saudi-Arabia	Apr.-2014	+	ND	NT
17-SAU-1	*L. vannamei*	Saudi-Arabia	Apr.-2015	+	ND	NT

14-NZL-1	*L. vannamei*	Newzealand	Apr.-2014	ND	NT	NT
15-NZL-1	*L. vannamei*	Newzealand	Apr.-2015	ND	NT	NT
**Domestic shellfish**						
11-BS-Oy-3	*Crassostrea gigas*	Busan	Mar.-2011	ND	NT	NT
11-BS-Oy-8	*C. gigas*	Busan	Aug.-2011	ND	NT	NT
11-BS-Oy-10	*C. gigas*	Busan	Oct.-2011	ND	NT	NT
11-BS-Oy-11	*C. gigas*	Busan	Nov.-2011	ND	NT	NT
12-BS-Oy-7	*C. gigas*	Busan	July-2012	ND	NT	NT
13-BS-Oy-2	*C. gigas*	Busan	Feb.-2013	ND	NT	NT
13-BS-Oy-3	*C. gigas*	Busan	Mar.-2013	ND	NT	NT
13-BS-Oy-4	*C. gigas*	Busan	Apr.-2013	ND	NT	NT
13-BS-Oy-5	*C. gigas*	Busan	May-2013	ND	NT	NT
13-BS-Oy-6	*C. gigas*	Busan	June-2013	ND	NT	NT
13-BS-Oy-7	*C. gigas*	Busan	July-2013	ND	NT	NT
13-BS-Oy-8	*C. gigas*	Busan	Aug.-2013	ND	NT	NT
13-BS-Oy-9 (1)	*C. gigas*	Busan	Sep.-2013	ND	NT	NT
13-BS-Oy-9 (2)	*C. gigas*	Busan	Sep.-2013	ND	NT	NT
13-BS-Oy-12	*C. gigas*	Busan	Dec.-2013	ND	NT	NT
14-BS-Oy-2	*C. gigas*	Busan	Feb.-2014	ND	NT	NT
14-BS-Oy-3	*C. gigas*	Busan	Mar.-2014	ND	NT	NT
14-BS-Oy-5	*C. gigas*	Busan	May-2014	ND	NT	NT
14-BS-Oy-6 (1)	*C. gigas*	Busan	June-2014	ND	NT	NT
14-BS-Oy-6 (2)	*C. gigas*	Busan	June-2014	ND	NT	NT
14-BS-Oy-8	*C. gigas*	Busan	Aug.-2014	ND	NT	NT
14-BS-Oy-9	*C. gigas*	Busan	Sep.-2014	ND	NT	NT
14-BS-Oy-10	*C. gigas*	Busan	Oct.-2014	+	ND	ND
14-BS-Oy-11 (1)	*C. gigas*	Busan	Nov.-2014	+	4.72×10^2	ND
14-BS-Oy-11 (2)	*C. gigas*	Busan	Nov.-2014	ND	NT	NT
11-DC-Oy-2	*C. gigas*	Daecheon	Feb.-2011	ND	NT	NT
11-DC-Oy-3	*C. gigas*	Daecheon	Mar.-2011	ND	NT	NT
11-DC-Oy-10	*C. gigas*	Daecheon	Oct.-2011	ND	NT	NT
12-DC-Oy-3	*C. gigas*	Daecheon	Mar.-2012	+	ND	13,046
13-DC-Oy-6	*C. gigas*	Daecheon	June-2013	ND	NT	ND
14-DC-Oy-4	*C. gigas*	Daecheon	Apr.-2014	ND	NT	NT
14-DC-Oy-5	*C. gigas*	Daecheon	May-2014	ND	NT	NT
14-DC-Oy-6	*C. gigas*	Daecheon	June-2014	ND	NT	NT
14-DC-Oy-7	*C. gigas*	Daecheon	July-2014	ND	NT	NT
14-DC-Oy-10	*C. gigas*	Daecheon	Oct.-2014	ND	NT	NT
14-DC-Oy-11	*C. gigas*	Daecheon	Nov.-2014	ND	NT	NT
14-DC-Oy-12	*C. gigas*	Daecheon	Dec.-2014	ND	NT	NT
11-DC-Mu-7	*Mytilus edulis*	Daecheon	July-2011	ND	NT	NT
11-DC-Mu-9	*M. edulis*	Daecheon	Sep.-2011	++	8.86×10^4	5,649
11-DC-Mu-10	*M. edulis*	Daecheon	Oct.-2011	ND	NT	NT
11-DC-Mu-11	*M. edulis*	Daecheon	Nov.-2011	++	1.64×10^4	ND
12-DC-Mu-5	*M. edulis*	Daecheon	May-2011	ND	NT	NT
12-DC-Mu-11	*M. edulis*	Daecheon	Nov.-2011	ND	NT	NT

13-DC-Mu-2	*M. edulis*	Daecheon	Feb.-2013	ND	NT	NT
13-DC-Mu-3	*M. edulis*	Daecheon	Mar.-2013	ND	NT	NT
13-DC-Mu-4	*M. edulis*	Daecheon	Apr.-2013	ND	NT	NT
13-DC-Mu-6	*M. edulis*	Daecheon	June-2013	ND	NT	NT
13-DC-Mu-7	*M. edulis*	Daecheon	July-2013	ND	NT	NT
13-DC-Mu-8	*M. edulis*	Daecheon	Aug.-2013	ND	NT	NT
13-DC-Mu-9	*M. edulis*	Daecheon	Sep.-2013	ND	NT	NT
13-DC-Mu-10	*M. edulis*	Daecheon	Oct.-2013	ND	NT	NT
13-DC-Mu-11	*M. edulis*	Daecheon	Nov.-2013	+	ND	ND
13-DC-Mu-12 (1)	*M. edulis*	Daecheon	Dec.-2013	ND	NT	NT
13-DC-Mu-12 (2)	*M. edulis*	Daecheon	Dec.-2013	ND	NT	NT
14-DC-Mu-1	*M. edulis*	Daecheon	Jan.-2014	ND	NT	NT
14-DC-Mu-2	*M. edulis*	Daecheon	Feb.-2014	ND	NT	NT
14-DC-Mu-3	*M. edulis*	Daecheon	Mar.-2014	ND	NT	NT
14-DC-Mu-4	*M. edulis*	Daecheon	Apr.-2014	ND	NT	NT
11-BS-Mu-6	*M. edulis*	Busan	June-2011	ND	NT	NT
11-BS-Mu-7	*M. edulis*	Busan	July-2011	ND	NT	NT
11-BS-Mu-8	*M. edulis*	Busan	Aug.-2011	ND	NT	NT
11-BS-Mu-9	*M. edulis*	Busan	Sep.-2011	ND	NT	NT
11-BS-Mu-10	*M. edulis*	Busan	Oct.-2011	ND	NT	NT
11-BS-Mu-11	*M. edulis*	Busan	Nov.-2011	ND	NT	NT
12-BS-Mu-9	*M. edulis*	Busan	Sep.-2012	++	4.71×10^4	5,783
13-BS-Mu-1	*M. edulis*	Busan	Jan.-2013	+	9.34×10^2	13,046
13-BS-Mu-2	*M. edulis*	Busan	Feb.-2013	ND	NT	NT
13-BS-Mu-3	*M. edulis*	Busan	Mar.-2013	ND	NT	NT
13-BS-Mu-4	*M. edulis*	Busan	Apr.-2013	ND	NT	NT
13-BS-Mu-7	*M. edulis*	Busan	July-2013	ND	NT	NT
13-BS-Mu-12	*M. edulis*	Busan	Dec.-2013	ND	NT	NT
14-BS-Mu-12	*M. edulis*	Busan	Dec.-2014	ND	NT	NT
11-GJ-Mu-4	*M. edulis*	Geoje	Apr.-2011	ND	NT	NT
11-GJ-Mu-5	*M. edulis*	Geoje	May-2011	ND	NT	NT
11-GJ-Mu-7	*M. edulis*	Geoje	July-2011	ND	NT	NT
11-GJ-Mu-10	*M. edulis*	Geoje	Oct.-2011	ND	NT	NT
13-GJ-Mu-2	*M. edulis*	Geoje	Feb.-2013	ND	NT	NT
14-GJ-Mu-2	*M. edulis*	Geoje	Feb.-2014	+	5.92×10^2	5,876
14-GJ-Mu-6	*M. edulis*	Geoje	June-2014	ND	NT	NT
14-GJ-Mu-9	*M. edulis*	Geoje	Sep.-2014	ND	NT	NT
12-TY-Mu-11	*M. edulis*	Tongyeong	Nov.-2012	ND	NT	NT
13-TY-Mu-8	*M. edulis*	Tongyeong	Aug.-2013	ND	NT	NT
14-TY-Mu-1	*M. edulis*	Tongyeong	Jan.-2014	ND	NT	NT
14-TY-Mu-2	*M. edulis*	Tongyeong	Feb.-2014	ND	NT	NT
14-TY-Mu-3	*M. edulis*	Tongyeong	Mar.-2014	ND	NT	NT
14-TY-Mu-10	*M. edulis*	Tongyeong	Oct.-2014	ND	NT	NT
14-TY-Mu-11	*M. edulis*	Tongyeong	Nov.-2014	ND	NT	NT
11-BS-Mc-6	*Venerupis philippinarum*	Busan	June-2011	ND	NT	NT
11-BS-Mc-9	*V. philippinarum*	Busan	Sep.-2011	ND	NT	NT

11-BS-Mc-10	*V. philippinarum*	Busan	Oct.-2011	ND	NT	NT
11-BS-Mc-11	*V. philippinarum*	Busan	Nov.-2011	ND	NT	NT
11-BS-Mc-12	*V. philippinarum*	Busan	Dec.-2011	ND	NT	NT
13-BS-Mc-1	*V. philippinarum*	Busan	Jan.-2013	ND	NT	NT
13-BS-Mc-2	*V. philippinarum*	Busan	Feb.-2013	+	2.27×10^3	777
13-BS-Mc-3	*V. philippinarum*	Busan	Mar.-2013	ND	NT	NT
13-BS-Mc-4	*V. philippinarum*	Busan	Apr.-2013	ND	NT	NT
13-BS-Mc-7	*V. philippinarum*	Busan	July-2013	ND	NT	NT
13-BS-Mc-8	*V. philippinarum*	Busan	Aug.-2013	ND	NT	NT
14-BS-Mc-1	*V. philippinarum*	Busan	Jan.-2014	ND	NT	NT
14-BS-Mc-2	*V. philippinarum*	Busan	Feb.-2014	+	ND	ND
14-BS-Mc-3	*V. philippinarum*	Busan	Mar.-2014	ND	NT	NT
14-BS-Mc-11	*V. philippinarum*	Busan	Nov.-2014	ND	NT	NT
11-SS-Mc-9	*V. philippinarum*	Seosan	Sep.-2011	++	2.54×10^4	ND
13-SS-Mc-1	*V. philippinarum*	Seosan	Jan.-2013	ND	NT	NT
13-SS-Mc-3 (1)	*V. philippinarum*	Seosan	Mar.-2013	ND	NT	NT
13-SS-Mc-3 (2)	*V. philippinarum*	Seosan	Mar.-2013	ND	NT	NT
13-SS-Mc-4	*V. philippinarum*	Seosan	Apr.-2013	ND	NT	NT
14-SS-Mc-10 (1)	*V. philippinarum*	Seosan	Oct.-2014	+	ND	ND
14-SS-Mc-10 (2)	*V. philippinarum*	Seosan	Oct.-2014	ND	NT	NT
14-SS-Mc-11	*V. philippinarum*	Seosan	Nov.-2014	ND	NT	NT
11-BG-Ga-6	*Tegillarca garnosa*	Beolgyo	June-2011	ND	NT	NT
11-BG-Ga-9 (1)	*T. garnosa*	Beolgyo	Sep.-2011	+	ND	ND
11-BG-Ga-9 (2)	*T. garnosa*	Beolgyo	Sep.-2011	ND	NT	NT
11-BG-Ga-9 (3)	*T. garnosa*	Beolgyo	Sep.-2011	ND	NT	NT
11-BG-Ga-10	*T. garnosa*	Beolgyo	Oct.-2011	ND	NT	NT
11-BG-Ga-11	*T. garnosa*	Beolgyo	Nov.-2011	ND	NT	NT
11-BG-Ga-12	*T. garnosa*	Beolgyo	Dec.-2011	ND	NT	NT
13-BG-Ga-1	*T. garnosa*	Beolgyo	Jan.-2013	+	ND	ND
13-BG-Ga-2	*T. garnosa*	Beolgyo	Feb.-2013	+	ND	ND
13-BG-Ga-3	*T. garnosa*	Beolgyo	Mar.-2013	ND	NT	NT
13-BG-Ga-4	*T. garnosa*	Beolgyo	Apr.-2013	ND	NT	NT
13-BG-Ga-8	*T. garnosa*	Beolgyo	Aug.-2013	ND	NT	NT
14-BG-Ga-1	*T. garnosa*	Beolgyo	Jan.-2014	+	ND	5,649
14-BG-Ga-2	*T. garnosa*	Beolgyo	Feb.-2014	ND	NT	NT
11-BA-Vc-7	*Mercenaria mercenaria*	Buan	July-2011	+	ND	ND
12-BA-Vc-2	*M. mercenaria*	Buan	Feb.-2012	++	9.83×10^3	11,070
12-BA-Vc-12	*M. mercenaria*	Buan	Dec.-2012	ND	NT	NT
13-BA-Vc-1	*M. mercenaria*	Buan	Jan.-2013	ND	NT	NT
13-BA-Vc-2	*M. mercenaria*	Buan	Feb.-2013	ND	NT	NT
13-BA-Vc-3	*M. mercenaria*	Buan	Mar.-2013	ND	NT	NT
13-BA-Vc-4	*M. mercenaria*	Buan	Apr.-2013	ND	NT	NT
13-BA-Vc-5	*M. mercenaria*	Buan	May-2013	ND	NT	NT
13-BA-Vc-8	*M. mercenaria*	Buan	Aug.-2013	+	ND	ND
14-BA-Vc-1 (1)	*M. mercenaria*	Buan	Jan.-2014	ND	NT	NT
14-BA-Vc-1 (2)	*M. mercenaria*	Buan	Jan.-2014	ND	NT	NT

14-BA-Vc-11	*M. mercenaria*	Buan	Nov.-2014	ND	NT	NT
11-MS-Oc-6	*Meretrix meretrix*	Masan	June-2011	ND	NT	NT
11-MS-Oc-9	*M. meretrix*	Masan	Sep.-2011	ND	NT	NT
11-MS-Oc-11	*M. meretrix*	Masan	Nov.-2011	ND	NT	NT
13-MS-Oc-10	*M. meretrix*	Masan	Sep.-2013	+	ND	ND
13-MS-Oc-12	*M. meretrix*	Masan	Dec.-2013	ND	NT	NT
11-TY-Sc-5	*Patinopecten yessoensis*	Tongyeong	May-2011	ND	NT	NT
11-TY-Sc-6	*P. yessoensis*	Tongyeong	June-2011	ND	NT	NT
13-TY-Sc-2	*P. yessoensis*	Tongyeong	Feb.-2013	+	ND	ND
13-TY-Sc-5	*P. yessoensis*	Tongyeong	May-2013	ND	NT	NT
13-TY-Sc-12	*P. yessoensis*	Tongyeong	Dec.-2013	ND	NT	NT
14-TY-Sc-3	*P. yessoensis*	Tongyeong	Mar.-2014	+	ND	ND
**Imported shellfish – China**						
11-CHN-Mc-1 (1)	*V. philippinarum*	China	Jan.-2011	ND	NT	NT
11-CHN-Mc-1 (2)	*V. philippinarum*	China	Jan.-2011	ND	NT	NT
11-CHN-Mc-3 (1)	*V. philippinarum*	China	Mar.-2011	ND	NT	NT
11-CHN-Mc-3 (2)	*V. philippinarum*	China	Mar.-2011	ND	NT	NT
11-CHN-Mc-11 (1)	*V. philippinarum*	China	Nov.-2011	ND	NT	NT
11-CHN-Mc-11 (2)	*V. philippinarum*	China	Nov.-2011	ND	NT	NT
11-CHN-Mc-11 (3)	*V. philippinarum*	China	Nov.-2011	ND	NT	NT
11-CHN-Mc-11 (4)	*V. philippinarum*	China	Nov.-2011	ND	NT	NT
12-CHN-Mc-3 (1)	*V. philippinarum*	China	Mar.-2012	ND	NT	NT
12-CHN-Mc-3 (2)	*V. philippinarum*	China	Mar.-2012	ND	NT	NT
12-CHN-Mc-11	*V. philippinarum*	China	Nov.-2012	ND	NT	NT
13-CHN-Mc-11 (1)	*V. philippinarum*	China	Nov.-2013	ND	NT	NT
13-CHN-Mc-11 (2)	*V. philippinarum*	China	Nov.-2013	ND	NT	NT
11-CHN-Vc-1	*M. mercenaria*	China	Jan.-2011	ND	NT	NT
11-CHN-Vc-6	*M. mercenaria*	China	June-2011	ND	NT	NT
11-CHN-Vc-11	*M. mercenaria*	China	Nov.-2011	ND	NT	NT
12-CHN-Vc-11	*M. mercenaria*	China	Nov.-2012	ND	NT	NT
13-CHN-Vc-3	*M. mercenaria*	China	Mar.-2013	++	4.17×10^4	ND
14-CHN-Vc-6	*M. mercenaria*	China	June-2014	ND	NT	NT
11-CHN-Oc-6	*M. meretrix*	China	June-2011	ND	NT	NT
12-CHN-Oc-3	*M. meretrix*	China	Mar.-2012	ND	NT	NT
14-CHN-Oc-8	*M. meretrix*	China	Aug.-2014	+	ND	ND
14-CHN-Oc-11	*M. meretrix*	China	Nov.-2014	ND	NT	NT
11-CHN-Cc-6	*C. sinensis*	China	June-2011	ND	NT	NT
12-CHN-Cc-3	*C. sinensis*	China	Mar.-2012	ND	NT	NT
13-CHN-Cc-3	*C. sinensis*	China	Mar.-2013	+	ND	ND
14-CHN-S-6	*Pationopecten yessoensis*	China	June-2014	ND	NT	NT
14-CHN-S-11	*P. yessoensis*	China	Nov.-2014	ND	NT	NT
11-CHN-Pc-3	*Saxidomus purpurata*	China	Mar.-2011	ND	NT	NT
14-CHN-Bsc-11	*Glycymeris vestita*	China	Nov.-2014	ND	NT	NT
11-JPN-Ga-4	*T. granosa*	Japan	Apr.-2011	ND	NT	NT
12-JPN-Ga-3 (1)	*T. granosa*	Japan	Mar.-2012	ND	NT	NT
12-JPN-Ga-3 (2)	*T. granosa*	Japan	Mar.-2012	ND	NT	NT

12-JPN-Ga-4	*T. granosa*	Japan	Apr.-2012	ND	NT	NT
14-JPN-Ga-1	*T. granosa*	Japan	Jan.-2014	ND	NT	NT
14-JPN-Ga-2	*T. granosa*	Japan	Feb.-2014	+	ND	NT
14-JPN-Ga-11 (1)	*T. granosa*	Japan	Nov.-2014	ND	NT	NT
14-JPN-Ga-11 (2)	*T. granosa*	Japan	Nov.-2014	ND	NT	NT

[a] Each shrimp group analyzed 5 individuals selected randomly of group, while each bivalve group included 3 individuals for analysis. [b]Determined based on the manufacturing (or import) date [c] ++: 1st PCR positive; +: 2nd PCR positive; If at least one individual in each group tests positive, the entire group is marked as positive. [d]Mean copies of positive samples (copies/mg)
